# Novel *Hawksworthiomyces* (*Ophiostomatales*, *Ophiostomataceae*) and *Melnikomyces* (*Venturiales*, *Sympoventuriaceae*) species and a new host record of *Hawksworthiomyces* on *Lindera aggregata* from China

**DOI:** 10.3897/mycokeys.139.197994

**Published:** 2026-09-02

**Authors:** Meng-Lan Chen, Nilita Mukjang, Xia Luo

**Affiliations:** 1 School of Biological and Food Engineering, Chuzhou University, Anhui, Chuzhou, 239000, China School of Biological and Food Engineering, Chuzhou University Chuzhou China; 2 Department of Biology, Faculty of Science, Chiang Mai University, Chiang Mai, 50200, Thailand Department of Biology, Faculty of Science, Chiang Mai University Chiang Mai Thailand

**Keywords:** Asexual morph, endophytic fungi, morphology, phylogeny, rhizosphere fungi, taxonomy

## Abstract

During a survey of root-associated fungal diversity of the medicinal plant *Lindera aggregata* in Anhui Province, China, three noteworthy fungal taxa belonging to *Hawksworthiomyces* and *Melnikomyces* were identified. Morphological and multilocus phylogenetic analyses revealed two previously undescribed species and one new host record. *Hawksworthiomyces rhizosphaerae***sp. nov**. and *Melnikomyces linderae***sp. nov**. are hereby described as new to science, while *H. taylorii* is reported for the first time from the roots of *L. aggregata*. *Hawksworthiomyces rhizosphaerae* is characterized by polyblastic conidiogenous cells bearing short cylindrical denticles and producing aseptate, hyaline, pyriform to ellipsoidal conidia, with secondary conidia frequently observed. *Melnikomyces linderae* is distinguished by pale brown to brown conidiophores, polyblastic conidiogenous cells with cylindrical denticles, verrucose 1(–2)-septate conidia released by rhexolytic secession, and globose to subglobose chlamydospores. Detailed descriptions and illustrations of these taxa are provided. These findings expand the known diversity of *Hawksworthiomyces* and *Melnikomyces* and contribute to the understanding of the root-associated fungal communities of *L. aggregata* in China.

## Introduction

*Hawksworthiomyces* was introduced by De Beer et al. (2016) within the family *Ophiostomataceae (Sordariomycetes)*, with *H. lignivorus* as the type species. Currently, eight species are listed in Index Fungorum (https://www.indexfungorum.org, accessed 18 June 2026). Members of this genus are characterized by mononematous, micronematous to macronematous conidiophores. The conidiogenous cells are polyblastic, integrated or discrete, and terminal or intercalary, with the apical part bearing denticles. The conidia are hyaline, aseptate, and ellipsoidal to cylindrical, with secondary conidia occasionally observed (De Beer et al. 2016). The sexual morph of *Hawksworthiomyces* remains unknown (Liu et al. 2025).

*Melnikomyces* was introduced by Crous et al. (2014) with the type species *M. vietnamensis*, which was collected from dry leaves in Vietnam. *Melnikomyces* was initially treated as *incertae sedis* within *Chaetothyriales (Eurotiomycetes)*. Hernández-Restrepo et al. (2020) resolved the taxonomic placement of *Melnikomyces* within the family *Sympoventuriaceae* (*Venturiales*, *Dothideomycetes*) through morphological and phylogenetic analyses. Currently, three species of *Melnikomyces* are accepted, including *M. longisporus*, *M. thailandicus*, and *M. vietnamensis* (Index Fungorum, https://www.indexfungorum.org, accessed 18 June 2026). The genus is characterized by brown, multiseptate, subcylindrical, erect to flexuous conidiophores. These conidiophores bear polyblastic conidiogenous cells that are terminal or intercalary, subcylindrical to subclavate, brown, and form a rachis with numerous denticle-like loci. Conidia are solitary, brown, verruculose, 1-septate, and fusoid-ellipsoidal with subobtuse ends and are released by rhexolytic secession. Additionally, the genus can produce terminal, smooth, brown, globose to subglobose chlamydospores arranged in short chains (Hernández-Restrepo et al. 2020; Wei et al. 2020).

Endophytic and rhizosphere fungi are important components of plant-associated microbial communities and can influence host growth, stress tolerance, nutrient acquisition, and secondary metabolite production (Aamir et al. 2020; Hashem et al. 2023; Ma et al. 2023; Hong et al. 2024). *Lindera aggregata* (*Lauraceae*) is an important medicinal plant widely used in traditional Chinese medicine (Chinese Pharmacopoeia Commission 2020). Although its phytochemistry and pharmacological activities have been extensively investigated (Lv et al. 2023; Wen et al. 2024), relatively little is known about the fungal communities associated with this host, with *Scytalidium linderae* being the only formally identified species from its roots to date (Chen et al. 2026). Characterizing the taxonomic diversity of root-associated fungi is an essential first step toward understanding their ecological functions and potential applications.

In this study, two novel species, *H. rhizosphaerae* sp. nov. and *M. linderae* sp. nov., were identified and described, and a new host record of *H. taylorii* was documented. These findings expand the known fungal diversity associated with this medicinal plant and provide a foundation for future studies on plant-fungus interactions, ecological functions, and the metabolic potential of root-associated fungi.

## Material and methods

### Collection and isolation

Healthy individuals of *L. aggregata* were collected from wild populations in natural subtropical evergreen broad-leaved mountainous forests of Anhui Province, China, at two sites in Huangshan City: Shexian County (29°52.77'N, 118°36.43'E, altitude 105 m) on 5 July 2023 and Huangshan District (30°6.09'N, 118°13.19'E, altitude 290 m) on 8 August 2023. The aboveground parts were removed prior to processing, and roots were gently shaken manually under sterile conditions to remove loosely attached soil. Subsequently, the roots were cut into small fragments using sterile scissors and placed into centrifuge tubes containing sterile 10 mM phosphate-buffered saline (PBS). The tubes were shaken at 120 rpm for 20 min at room temperature to separate the soil closely associated with the roots. Following this, the root tissues were removed and collected as root samples, while the resulting suspension was centrifuged at 8,000 rpm for 20 min at 4 °C to obtain the rhizosphere soil.

Rhizosphere microorganisms were isolated using the serial dilution method (Zheng et al. 2019). Briefly, 10 g of soil was suspended in 90 mL of sterile distilled water and agitated for 30 min to prepare the 10^−1^ (w/v) stock. A tenfold serial dilution was then performed to reach a final concentration of 10^−6^. Subsequently, 100 μL aliquots from each dilution (10^−2^ to 10^−6^) were spread onto potato dextrose agar (PDA) plates. All plates were incubated at 28 °C for 2–7 days.

Root samples were processed according to a strict surface-sterilization protocol (Zhang et al. 2023), with some modifications. The roots were first washed under running tap water and then transferred to a laminar flow cabinet. The tissues were soaked in 75% ethanol for 3 min, rinsed with sterile water, and then treated with 10% sodium hypochlorite for 2–3 min. After three final rinses in sterile water to remove any residues, the roots were dried on sterile filter paper and cut into small pieces. To verify the effectiveness of sterilization, the final rinse water was spread onto PDA plates as a control. No microbial growth was observed on the control plates. All samples and control plates were incubated at 28 °C for 2–7 days, after which individual colonies were picked and purified for further study.

### Morphological study

For morphological characterization, the strains were cultured on five media: PDA, malt extract agar (MEA), cornmeal agar (CMA), oatmeal agar (OA), and water agar (WA). Colony features, including size, shape, color, and texture, were recorded. Observations and measurements of micromorphological structures were performed with a NIKON ECLIPSE Ni-U compound microscope coupled with a DS-Ri2 digital camera and Tarosoft (R) Image Frame Work software (v.0.9.7). Digital images were processed and assembled using Adobe Photoshop v.19.1.6 (Adobe Systems, USA). Voucher specimens were deposited in HKAS, and living cultures were preserved in KUNCC.

### Molecular identification

Genomic DNA was extracted from fresh fungal mycelia using the Genomic DNA Extraction Kit (BioFlux, Shanghai, China). The fungal strains were cultivated on PDA at 25 °C for 2 weeks. The internal transcribed spacer (ITS), the large subunit nuclear ribosomal DNA (LSU), the small subunit of nuclear ribosomal RNA (SSU), the RNA polymerase II second-largest subunit (*RPB2*), translation elongation factor 1-alpha (*TEF1-α*), beta-tubulin (*TUB2*), calmodulin (*CAL*), and actin (*ACT1*) genes were amplified and sequenced. Polymerase chain reaction (PCR) was conducted in a total volume of 25 μL containing 12.5 μL of 2× Taq PCR Master Mix, 9.5 μL of ddH_2_O, 0.5 μL of each forward and reverse primer, and 2 μL of DNA template. The primer pairs used for each locus were as follows: ITS1/ITS4 for ITS (White et al. 1990), LR0R/LR5 for LSU (Vilgalys and Hester 1990), NS1/NS4 for SSU (White et al. 1990), RPB2-5F/RPB2-7CR and RPB2AM-1bf/RPB2AM-7R for *RPB2* (Liu et al. 1999), TEF1-983F/EF1-1567R for *TEF1-α* (Carbone and Kohn 1999; Rehner and Buckley 2005), Bt2a/Bt2b for *TUB2* (Glass and Donaldson 1995), CL-2F/CL-2R for *CAL* (Duong et al. 2012), and ACT-512F/ACT-783R for *ACT1* (Carbone and Kohn 1999). The PCR thermal cycling conditions for each locus followed the protocols described in the corresponding references above. Subsequently, the PCR amplicons were purified and commercially sequenced by Sangon Biotech (Nanjing, China).

### Phylogenetic analysis

The raw sequences were quality-checked and assembled using BioEdit v.7.1.3.0 and SeqMan v.7.0.0 (DNASTAR, Madison, WI, USA), respectively (Hall 1999). To obtain preliminary taxonomic identification, the newly assembled sequences were subjected to BLAST searches in the NCBI GenBank database. Based on the BLAST results and relevant recent publications (Hernández-Restrepo et al. 2020; Wei et al. 2020; Crous et al. 2023), reference sequences for phylogenetic analyses were selected and retrieved from GenBank. Nucleotide sequences were edited in BioEdit, aligned with MAFFT, and then optimized with trimAl v.1.2 (Capella-Gutiérrez et al. 2009). Multigene alignments were combined using Sequence Matrix 1.7.8 (Vaidya et al. 2011). All sequences were submitted to GenBank, and their accession numbers are listed in Tables 1, 2.

**Table 1.** Taxa used in the phylogenetic analyses for the genus *Hawksworthiomyces* and their GenBank accession numbers.

**Species**	**Strain number**	**ITS**	**LSU**	***TEF1-α***	***RPB2***	***TUB2***	***CAL***
*Aureovirgo volantis*	CMW41250	OM501369	OM514700	OM631743	OM631579	KR051108	N/A
*Aureovirgo volantis*	CMW38929	OM501368	OM514699	OM631742	OM631578	N/A	N/A
*Ceratocystiopsis minima*	CMW162	OM501370	OM514701	OM631744	N/A	N/A	N/A
*Ceratocystiopsis minuta*	CMW4352	OM501372	OM514703	OM631745	OM631581	MW066756	N/A
*Ceratocystiopsis neglecta*	CMW22403	OM501373	OM514704	OM631746	OM631582	N/A	N/A
*Graphilbum crescericum*	CMW22828	OM501403	OM514749	OM631779	OM631604	N/A	N/A
*Graphilbum fragrans*	CMW19357	OM501404	OM514750	OM631780	OM631605	N/A	N/A
*Graphilbum ipis grandicollis*	VPRI43762	MW046071	MW046117	MW066405	N/A	MW066359	MW075120
*Graphilbum microcarpum*	CMW17196	OM501405	OM514751	OM631781	OM631606	N/A	N/A
*Graphilbum nigrum*	CMW1097	OM501406	OM514752	N/A	OM631607	N/A	N/A
*Graphilbum pusillum*	CMW14490	OM501407	OM514753	N/A	N/A	N/A	N/A
*Grosmannia pruni*	CMW10418	OM501398	OM514740	OM631775	N/A	N/A	N/A
*Grosmannia taigensis*	CMW36629	OM501400	OM514744	OM631777	OM631602	N/A	N/A
*Harringtonia aguacate*	Raff sp 272	MT633065	MT629748	OM631783	OM631613	N/A	N/A
*Harringtonia lauricola*	Raff sp 570	MT633071	MT629759	OM631784	OM631614	MT644093	N/A
*Hawksworthiomyces ciconiae*	CBS 147964	NR 190972	N/A	OR195097	N/A	OR195095	OR195096
*Hawksworthiomyces crousii*	OTU2029	MT595243	N/A	N/A	N/A	N/A	N/A
*Hawksworthiomyces crousii*	CMW 37531	KX396551	KX396548	OM652622	OM631609	N/A	N/A
*Hawksworthiomyces cylindroconidius*	GMBC5346	PV932973	PV932992	N/A	N/A	N/A	N/A
*Hawksworthiomyces cylindroconidius*	GMBC5345	PV932972	PV932991	N/A	N/A	N/A	N/A
*Hawksworthiomyces geerae*	BRIP 76881a	PX692687	PX914131	N/A	N/A	N/A	N/A
*Hawksworthiomyces hibbettii*	CMW37663	KX396550	KX396547	OM652623	OM631610	N/A	N/A
*Hawksworthiomyces lignivorus*	CMW18600	OM501410	OM514756	OM631782	OM631611	N/A	N/A
*Hawksworthiomyces riparius*	FMR19083	ON000432	ON000433	N/A	N/A	N/A	N/A
*Hawksworthiomyces taylorii*	CMW20741	KX396549	KX396546	OM652624	OM631612	N/A	N/A
***Hawksworthiomyces taylorii***	**KUNCC24-1761**4	**PZ331851**	**PZ326430**	**PZ575097**	**PZ575094**	**PZ575100**	**PZ575091**
***Hawksworthiomyces rhizosphaerae***	**KUNCC24-17610**	**PZ331849**	**PZ326428**	**PZ575095**	**PZ575092**	**PZ575098**	**PZ575089**
***Hawksworthiomyces rhizosphaerae***	**KUNCC25-20165**	**PZ331850**	**PZ326429**	**PZ575096**	**PZ575093**	**PZ575099**	**PZ575090**
*Heinzbutinia grandicarpa*	CMW1600	OM501412	OM514757	OM631786	OM631616	N/A	N/A
*Jamesreidia coronata*	CMW43836	OM501413	OM514759	OM631788	OM631618	N/A	N/A
*Jamesreidia nigrocarpa*	CMW651	AY280490	DQ294356	N/A	N/A	AY280480	N/A
*Jamesreidia tenella*	CMW37439	OM501414	OM514760	OM631789	N/A	N/A	N/A
*Leptographium bhutanense*	CMW18649	OM501418	OM514762	OM631794	N/A	EU650191	N/A
*Leptographium castellanum*	CMW2320	OM501420	OM514764	OM631798	OM631623	N/A	N/A
*Leptographium douglasii*	CMW2076	OM501424	OM514766	OM631802	OM631627	KY424512	KY424522
*Leptographium profanum*	CMW10552	OM501443	OM514779	OM631817	OM631640	N/A	N/A
*Leptographium wageneri*	CMW279	OM501458	OM514746	OM631831	OM631651	N/A	N/A
*Masuyamyces ambrosius*	CMW1024	OM501465	OM514793	OM631836	OM631656	N/A	N/A
*Masuyamyces botuliformis*	CMW14493	OM501471	OM514799	OM631837	N/A	N/A	N/A
*Masuyamyces saponiodorus*	CMW28135	OM501512	OM514838	OM631838	OM631657	N/A	N/A
*Ophiostoma ainoae*	CMW1037	OM501463	OM514791	N/A	N/A	HM031552	N/A
*Ophiostoma brunneolum*	CMW23145	OM501472	OM514800	OM631843	OM631663	N/A	N/A
*Ophiostoma clavatum*	CMW37983	OM501477	OM514805	OM631848	N/A	N/A	N/A
*Ophiostoma poligraphi*	CMW38899	OM501507	OM514832	OM631871	OM631688	N/A	N/A
*Ophiostoma tapionis*	CMW23266	OM501516	OM514842	OM631881	OM631697	HM031544	N/A
*Raffaelea ambrosiae*	CMW25533	MT633067	MT629751	OM631891	OM631706	EU977472	N/A
*Raffaelea canadensis*	CMW25536	GQ225699	MT629755	N/A	N/A	N/A	N/A
*Raffaelea cyclorhipidii*	CMW44790	MT633069	MT629757	N/A	OM631710	N/A	N/A
*Raffaelea xyleborini*	CMW45859	MT633078	MT629769	N/A	N/A	N/A	N/A
*Sporothrix aemulophila*	CMW40381	OM501527	OM514854	OM631897	N/A	N/A	N/A
*Sporothrix brasiliensis*	CMW29127	OM501531	OM514859	OM631901	N/A	N/A	N/A
*Sporothrix cabralii*	CMW38098	OM501533	OM514861	OM631903	OM631716	N/A	KX590804
*Sporothrix mexicana*	CMW29129	OM501547	OM514875	OM631919	N/A	N/A	N/A
*Sporothrix schenckii*	CMW38850	OM501560	OM514887	OM631928	OM631737	N/A	N/A
*Graphium carbonarium*	CBS 123611	MH863311	MH874835	N/A	N/A	N/A	N/A
*Parascedosporium putredinis*	CBS 133438	MH866067	MH877539	N/A	N/A	N/A	N/A

**Table 2.** Taxa used in the phylogenetic analyses for the genus *Melnikomyces* and their GenBank accession numbers.

**Species**	**Strain number**	**ITS**	**LSU**	**SSU**	***TUB2***	***TEF1-α***	***ACT1***
*Ochroconis constricta*	CBS 202 27	AB161063	KF156147	KF156072	KF156161	KF156003	KF155942
*Ochroconis anellii*	CBS 284 64	FR832477	KF156138	KF156070	KF156184	KF155995	KF155912
*Ochroconis bacilliformis*	CBS 100442	KP798632	KP798635	KP798638	KT272059	KT272070	KT272051
*Ochroconis phaeophora*	CBS 206 96	KP798631	KP798634	KP798637	KT272062	KT272098	KT272054
*Ochroconis tshawytschae*	CBS 100438	HQ667562	KF156126	KF156062	KF156180	KF155990	KF155918
*Verruconis calidifluminalis*	CBS 125818	AB385698	KF156108	KF156046	KF156202	KF155959	KF155901
*Verruconis gallopava*	CBS 437 64	HQ667553	KF156112	KF156053	KF156203	KF155968	HQ916989
*Verruconis verruculosa*	CBS 119775	KF156014	KF156106	KF156055	KF156193	KF155974	KF155919
*Verruconis pseudotricladiata*	YMF1 04915	MK244396	MK248270	MK248268	MK253013	MK248273	N/A
*Verruconis panacis*	CBS 142802	MF536882	MF536880	MF536879	MF536883	MF536881	N/A
*Scolecobasidium excentricum*	CBS 469 95	HQ667543	KF156105	KF156096	KF156196	KF155975	KF155934
*Clavatispora thailandica*	MFLUCC 100107	MH065721	KF770458	KF770457	N/A	N/A	N/A
*Sympoventuria capensis*	CBS 120136	KF156039	KF156104	KF156094	N/A	N/A	N/A
*Fusicladium sicilianum*	CBS 105 85	FN549914	FN398150	KP798640	N/A	N/A	N/A
*Mycosisymbrium cirrhosum*	MTCC 12435	KR259883	KR259884	KR259885	N/A	N/A	N/A
*Melnikomyces thailandicus*	CBS 145767	MN794374	MN794351	N/A	N/A	N/A	N/A
*Melnikomyces vietnamensis*	CBS 136209	KJ869156	KJ869213	N/A	N/A	N/A	N/A
*Melnikomyces longisporum*	HGUP 18226	MT731290	MT731291	MT731289	MT739515	MT739516	MT739517
*Melnikomyces longisporum*	6 ITS1	OQ519907	N/A	N/A	N/A	N/A	N/A
***Melnikomyces linderae***	**KUNCC24-17617**	**PZ326426**	**PZ326431**	**PZ326434**	**PZ341873**	**PZ341871**	**PZ575087**
***Melnikomyces linderae***	**KUNCC25-20169**	**PZ326427**	**PZ326432**	**PZ326435**	**PZ341874**	**PZ341872**	**PZ575088**
*Matsushimaea monilioides*	CBS 143867	LT883468	LT883469	N/A	N/A	N/A	N/A
*Apiosporina collinsii*	CBS 118973	EU035443	GU301798	GU296135	N/A	N/A	N/A
*Venturia populina*	CBS 256 38	EU035467	GU323212	GU296206	N/A	KF853986	N/A

Sequence alignments were exported in FASTA format for maximum likelihood (ML) analyses and in NEXUS format for Bayesian inference (BI) analyses (Larsson 2014). Phylogenetic analyses were performed on the CIPRES Science Gateway platform (Miller et al. 2010). For ML analysis, the RAxML-HPC v.8 tool with rapid bootstrap analysis was used, followed by 1,000 bootstrap replicates (Miller et al. 2010; Stamatakis 2014). The best-scoring ML tree was selected from the resulting topologies based on likelihood scores under the GTRGAMMA substitution model. BI was performed in MrBayes 3.2.7a (Ronquist et al. 2012), with the optimal nucleotide substitution model for each partition determined using MrModeltest v.2.3 (Nylander 2004). For *Melnikomyces*, GTR+I+G was estimated as the best-fit model for ITS, LSU, SSU, *TUB2*, and *ACT1*, and GTR+G for *TEF1-α*, according to the Akaike information criterion (AIC). For *Hawksworthiomyces*, GTR+I+G was estimated as the best-fit model for ITS, LSU, *TEF1-α*, *RPB2*, and *TUB2*, and GTR+G for *CAL*, under the same criterion. Four Markov chains were run for one million generations, and trees were sampled every 100 generations. The average standard deviation of split frequencies was < 0.01. A burn-in of 2,000 trees (20%) was discarded before calculating posterior probabilities from the remaining 8,000 trees.

Phylogenetic trees were visualized in FigTree v.1.2.2 (Rambaut and Drummond 2008), with the final layout prepared in Adobe Illustrator CS5 (Adobe Systems, USA). The alignments and phylogenetic trees for the genera *Hawksworthiomyces* and *Melnikomyces* were deposited in TreeBASE under accession numbers 32693 and 32692, respectively (http://www.treebase.org).

## Results

### Phylogenetic analysis

The phylogram of *Hawksworthiomyces* was generated from ML analysis based on concatenated six-locus sequence data comprising ITS, LSU, *TEF1-α*, *RPB2*, *TUB2*, and *CAL.* The best-scoring RAxML tree with a final likelihood value of −28349.261640 is presented. The matrix had 1,510 distinct alignment patterns, with 36.85% undetermined characters or gaps; the tree length was 3.350100. Estimated base frequencies were as follows: A = 0.223373, C = 0.283644, G = 0.301750, and T = 0.191233, with substitution rates AC = 1.263388, AG = 2.591401, AT = 1.212630, CG = 2.031486, CT = 6.598259, and GT = 1.000000; GAMMA distribution shape parameter *α* = 0.221736. The final average standard deviation of split frequencies was 0.009456.

**Figure 1.**
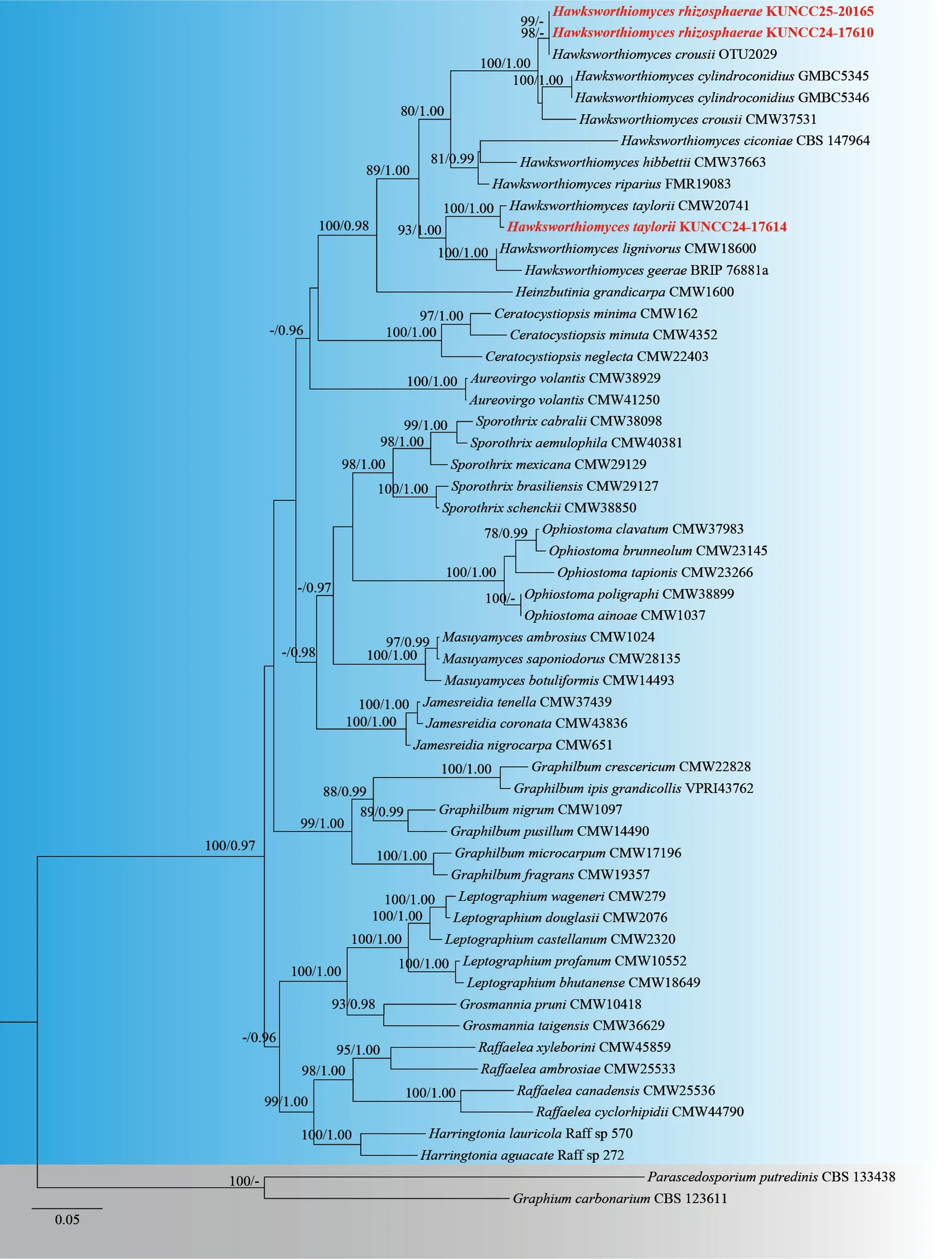
Phylogram from ML analysis based on combined ITS, LSU, *TEF1-α*, *RPB2*, *TUB2*, and *CAL* sequence data. MLBS values equal to or greater than 75% and BYPP values equal to or greater than 0.95 are given at the nodes (MLBS/BYPP). The tree is rooted with *Parascedosporium putredinis* (CBS 133438) and *Graphium carbonarium* (CBS 123611). Species names and culture collection numbers in red indicate newly collected taxa.

Phylogenetic analyses positioned two newly collected taxa within *Hawksworthiomyces* (Fig. 1). Combined ITS, LSU, *TEF1-α*, *RPB2*, *TUB2*, and *CAL* phylogenetic analyses confirmed the placement of *H. rhizosphaerae* (KUNCC 24-17610 and KUNCC 25-20165) in a clade with *H. crousii* (OTU2029). This clade formed a distinct and well-supported lineage (100% maximum likelihood bootstrap support [MLBS]/1.00 Bayesian posterior probability [BYPP]) that is sister to another clade containing *H. crousii* (CMW 37531) and *H. cylindroconidius* (GMBC5345 and GMBC5346). Another isolate, *H. taylorii* (KUNCC24-17614), grouped with the ex-holotype strain of *H. taylorii* (CMW20741) with strong support values (100% MLBS/1.00 BYPP), indicating that they represent the same species.

The phylogram of *Melnikomyces* was generated from ML analysis based on concatenated six-locus sequence data comprising ITS, LSU, SSU, *TUB2*, *TEF1-α*, and *ACT1*. The best-scoring RAxML tree with a final likelihood value of −22664.878237 is presented. The matrix had 1,358 distinct alignment patterns, with 21.78% undetermined characters or gaps; the tree length was 2.650997. Estimated base frequencies were as follows: A = 0.241616, C = 0.245852, G = 0.283568, and T = 0.228964, with substitution rates AC = 1.489814, AG = 2.608865, AT = 1.463032, CG = 1.312431, CT = 5.296508, and GT = 1.000000; GAMMA distribution shape parameter *α* = 0.222892. The final average standard deviation of split frequencies converged to 0.009647.

**Figure 2.**
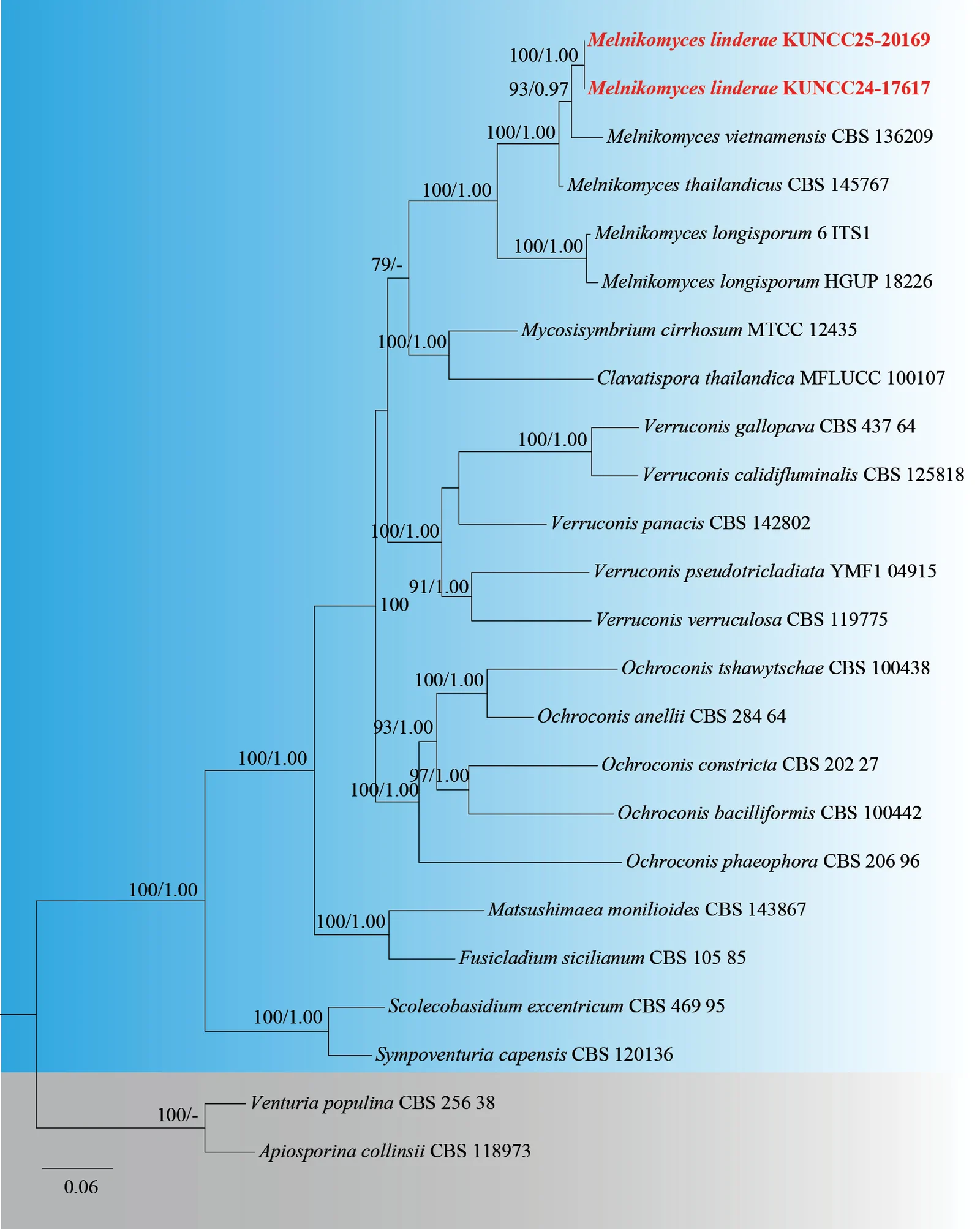
Phylogram from ML analysis based on combined ITS, LSU, SSU, *TUB2*, *TEF1-α*, and *ACT1* sequence data. MLBS values equal to or greater than 75% and BYPP values equal to or greater than 0.95 are given at the nodes (MLBS/BYPP). The tree is rooted with *Apiosporina collinsii* (CBS 118973) and *Venturia populina* (CBS 256 38). The isolate from the current study is highlighted in red.

Phylogenetic analyses positioned the newly collected taxon within the genus *Melnikomyces* (Fig. 2). Multilocus phylogeny showed that the new species *M. linderae* (KUNCC24-17617 and KUNCC25-20169) formed a distinct clade, strongly supported as sister to *M. vietnamensis* (CBS 136209) (93% MLBS/0.97 BYPP), supporting its recognition as a novel species.

### Taxonomy

#### Hawksworthiomyces rhizosphaerae

Taxon classificationFungiOphiostomatalesOphiostomataceae

M.L. Chen & X. Luo sp. nov.

CC2B9D8D-2F75-537C-B329-AA25DE90D6C5

Index Fungorum: IF905462

Fig. 3

##### Etymology.

The epithet “*rhizosphaerae*” refers to the rhizosphere soil of *L. aggregata*, from which the strain was isolated.

##### Holotype.

HKAS 133196.

##### Description.

**Sexual morph** not observed. **Asexual morph: *Conidiophores*** mononematous, micronematous or semi-macronematous, cylindrical, straight or flexuous, hyaline, sparingly branched, smooth-walled, reduced to conidiogenous cells. ***Conidiogenous cells*** 1.2–25.5 μm long, 0.5–1.1 μm wide at base, polyblastic, integrated or discrete, terminal or intercalary, cylindrical, straight or curved, hyaline, sympodial, ending in fertile region with multiple denticles, occasionally swollen apex. ***Denticles*** distinct, short-cylindrical, 0.3–0.6 × 0.3–0.5 μm. ***Conidia*** 0-septate, diverse in shape, varying from pyriform, cylindrical, broadly ellipsoidal to ellipsoidal, tapering towards the base, base truncate, sometimes formed singly on denticles directly emerging from vegetative hyphae, producing secondary conidia, hyaline, guttulate, (1.6–)1.8–3.9 × 0.8–2.1 μm.

**Figure 3.**
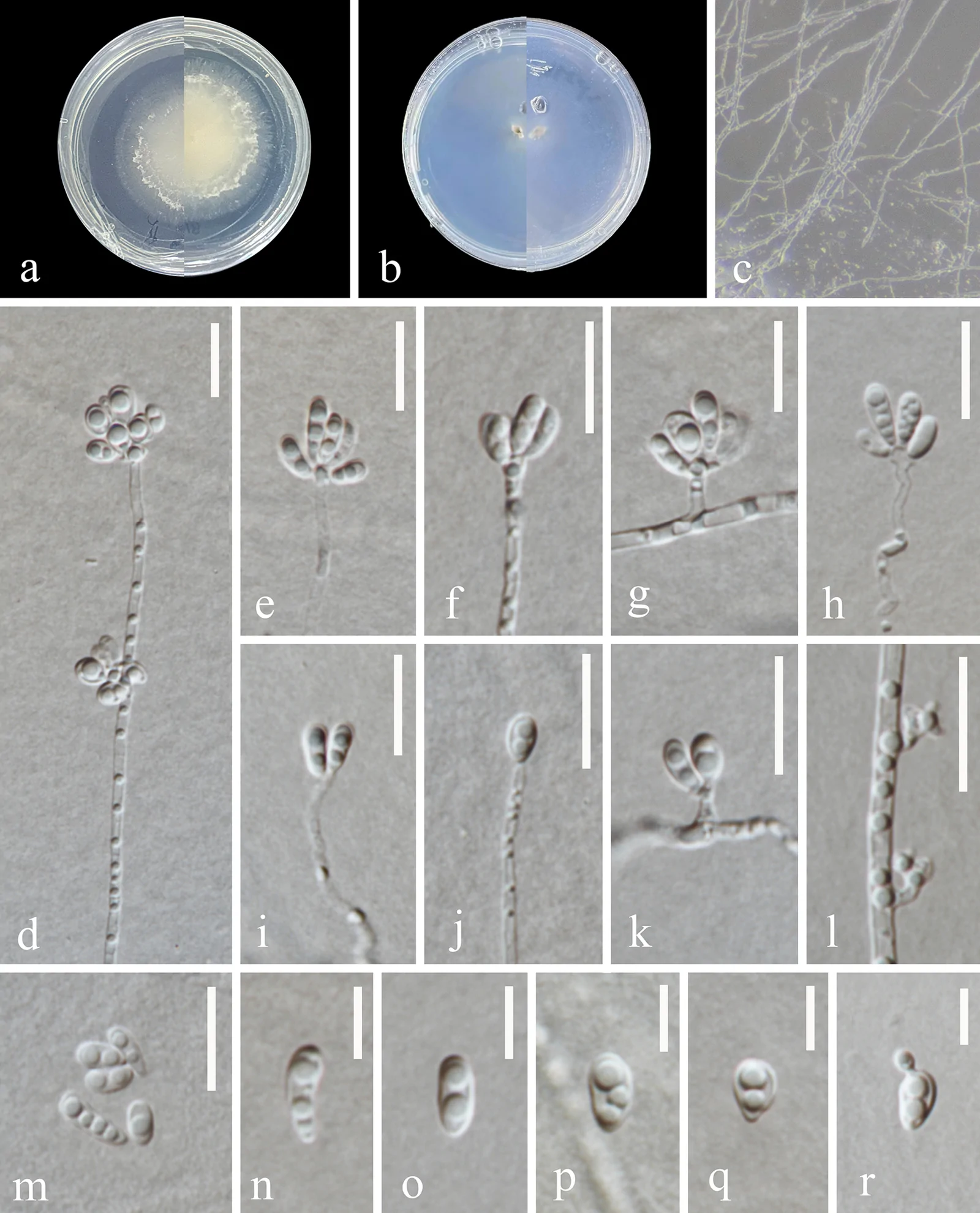
*Hawksworthiomyces rhizosphaerae* sp. nov. (HKAS 133196, holotype). **a, b**. Cultures on PDA and WA from above and below; **c**. Colonies on WA; **d–l**. Conidiogenous cells and conidia; **m–q**. Conidia; **r**. Secondary conidia. Scale bars: 10 μm (**d–m**); 5 μm (**n–r**).

##### Culture characteristics.

Colonies growing on PDA reached 3.2 cm in diam. after 2 weeks at 28 °C, circular, flat, moist, regular edge, milky white; white to milky white in reverse. Colonies on WA at 28 °C exhibit thin mycelia with sporulation, slow growth, and white from both above and reverse.

##### Material examined.

China • Anhui Province, Huangshan City, She County, 29°52.77'N, 118°36.43'E, elevation 105 m, rhizosphere soil of *L. aggregata*, 5 July 2023, Meng-Lan Chen, SP2-16 (Holotype: HKAS 133196, ex-type culture: KUNCC24-17610, ex-isotype culture: KUNCC25-20165).

##### Notes.

*Hawksworthiomyces rhizosphaerae* is characterized by polyblastic, sympodial conidiogenous cells (1.2–25.5 × 0.5–1.1 μm) bearing distinct denticles and hyaline, guttulate, aseptate, pyriform to ellipsoidal conidia (1.8–3.9 × 0.8–2.1 μm). Phylogenetically, *H. rhizosphaerae* is closely related to *H. crousii* and *H. cylindroconidius* (Fig. 1). *Hawksworthiomyces rhizosphaerae* (KUNCC24-17610) differs from *H. crousii* (CMW 37531) in 8/793 bp (1.01%) of LSU, 15/480 bp (3.13%) of ITS, 4/226 bp (1.77%) of *TEF1-α*, and 25/534 bp (4.68%) of *RPB2*; and from *H. cylindroconidius* (GMBC5345) in 5/793 bp (0.63%) of LSU and 8/449 bp (1.78%) of ITS. *Hawksworthiomyces crousii* (CMW 37531) differs from *H. rhizosphaerae* (KUNCC24-17610) in having slightly wider conidiogenous cells (5–22 × 1–2 μm) and larger denticles (0.5–1 × 0.5–1 μm vs. 0.3–0.6 × 0.3–0.5 μm). Furthermore, the conidia of *H. crousii* are larger (3–6.5 × 1.5–3.5 μm) than those of *H. rhizosphaerae* and are not distinctly guttulate (De Beer et al. 2016). *Hawksworthiomyces cylindroconidius* (GMBC5345) differs from *H. rhizosphaerae* (KUNCC24-17610) in having monoblastic conidiogenous cells and significantly longer, unbranched conidiophores (94.5–131.5 × 11–20.5 μm), whereas *H. rhizosphaerae* possesses polyblastic conidiogenous cells with distinct denticles (Liu et al. 2025). Therefore, based on morphological differences and phylogenetic evidence, *H. rhizosphaerae* is introduced herein as a novel species.

#### Hawksworthiomyces taylorii

Taxon classificationFungiOphiostomatalesOphiostomataceae

Z.W. de Beer, Marinc., M.J. Wingf.

4056D7B8-3CF1-5C3C-9258-42A3F19B102D

Index Fungorum: IF815687

Fig. 4

##### Description.

**Sexual morph** not observed. **Asexual morph: *Conidiophores*** mononematous, macronematous, cylindrical, straight or flexuous, hyaline to light brown, branched, reduced to conidiogenous cells. ***Conidiogenous cells*** (2.1–)3.3–19.7 × 0.8–1.7 μm, polyblastic, integrated or discrete, terminal or intercalary, cylindrical, slightly tapering toward apex, fertile region with denticles. ***Denticles*** diminutive, conical. ***Conidia*** aseptate, subglobose, cylindrical to ellipsoidal, tapering towards base, base truncated, smooth, hyaline, guttulate, 1.3–3.8 × 0.9–1.9 μm.

**Figure 4.**
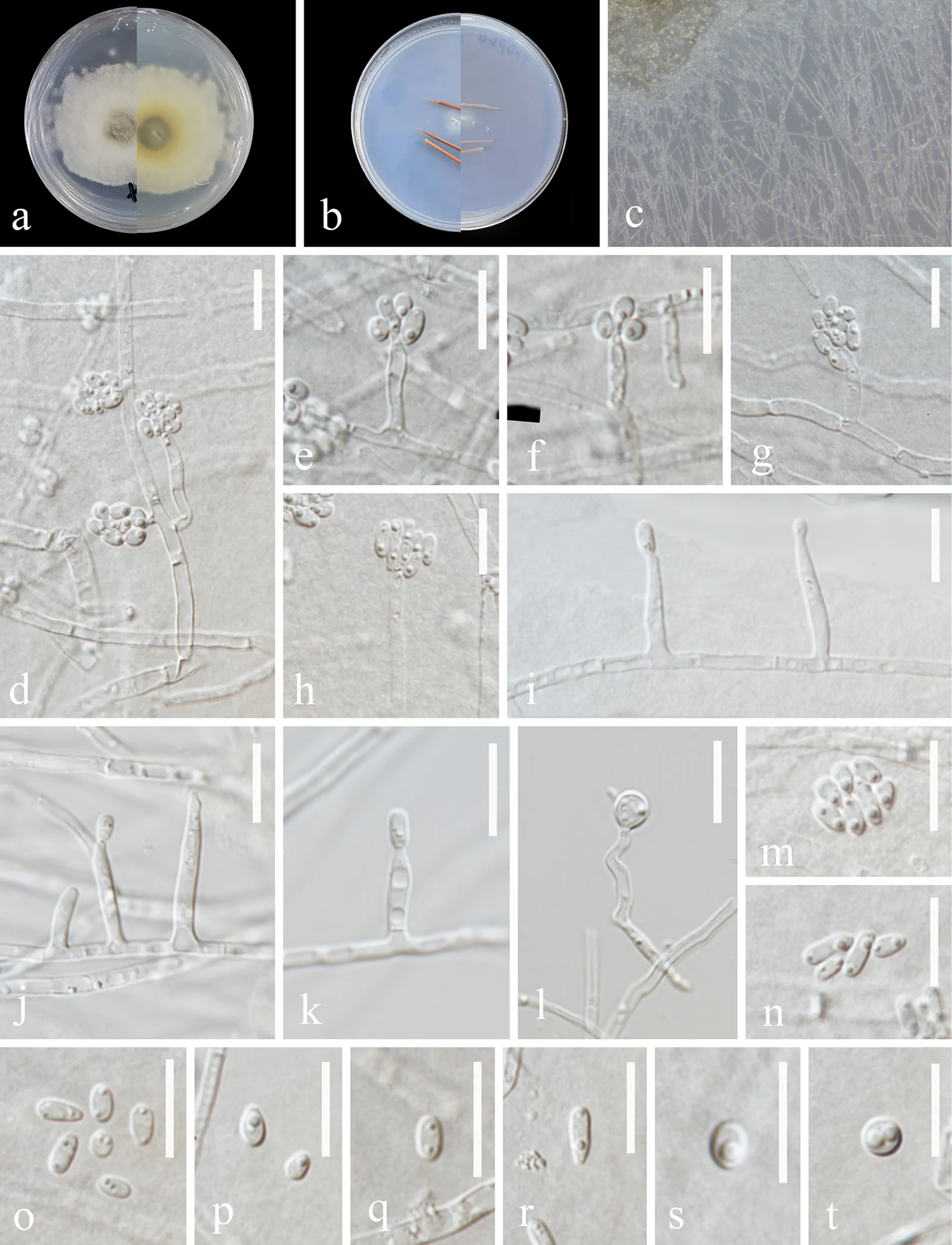
*Hawksworthiomyces taylorii* (HKAS 133197, new host record). **a, b**. Cultures on PDA and WA from above and below; **c**. Colonies on WA; **d–l**. Conidi ogenous cells and conidia; **m–t**. Conidia. Scale bars: 10 μm (**d–t**).

##### Culture characteristics.

On PDA, colonies grew to 3.5 cm in diameter after 2 weeks at 28 °C, circular, flat, dense, velvety to cottony, white to dusky yellow-green. Reverse olive green at the center, white to light yellow toward the margin. On WA, colonies were slow growth and white on both the above and reverse.

##### Known distribution and hosts.

*Eucalyptus* utility pole at soil level in South Africa (De Beer et al. 2016); adult beetles of *Hylurgus ligniperda* in China (Xie et al. 2025); the gut of the fungus-growing termite *Macrotermes barneyi* in China (Nagam et al. 2021); soil in China (Zhang et al. 2022); and the roots of *L. aggregata* in China (this study).

##### Material examined.

China • Anhui Province, Huangshan City, Huangshan District, 30°6.09'N, 118°13.19'E, elevation 290 m, root of *L. aggregata*, 8 August 2023, Meng-Lan Chen, TKDP3-4 (Specimen examined: HKAS 133197, living culture: KUNCC24-17614).

##### Notes.

Phylogenetically (Fig. 1), the collection in this study clustered with the ex-holotype strain of *H. taylorii* (CMW20741). *Hawksworthiomyces taylorii* was originally introduced by De Beer et al. (2016) from a *Eucalyptus* utility pole in South Africa. Morphologically, the newly collected strain had morphology similar to that of *H. taylorii* (ex-type strain, CMW20741) (De Beer et al. 2016). This study reports a new host record of *H. taylorii* from the medicinal plant *L. aggregata*.

#### Melnikomyces linderae

Taxon classificationFungiChaetothyriales

M.L. Chen & X. Luo sp. nov.

4B204CFE-C086-59D0-9522-002853E56A40

Index Fungorum: IF905463

Fig. 5

##### Etymology.

The epithet “*linderae*” refers to the host genus *Lindera*, from which the fungus was originally isolated.

##### Holotype.

HKAS 133201.

##### Description.

**Sexual morph** not observed. **Asexual morph: *Conidiophores*** 8.2–46.0 × 0.9–1.7 μm, macronematous, unbranched or branched, thick and smooth-walled, erect, straight or slightly flexuous, septate, subcylindrical, light brown to brown, usually paler towards apex, reduced to conidiogenous cells. ***Conidiogenous cells*** 3.5–15.9 × 0.7–1.5 μm, polyblastic, integrated, terminal or intercalary, pale brown to brown, sympodial, with one or more denticles in the apical region, denticles cylindrical, pale brown, 0.4–1.0 μm long. ***Conidia*** 3.0–6.4 × 1.8–2.7 μm, solitary, ellipsoidal to obovoid, occasionally slightly clavate, aseptate when young, 1-septate when mature, sometimes 2-septate, slightly constricted at the septum, hyaline to light brown, verrucose, rhexolytic secession. ***Chlamydospores*** 3.6–7.6 × 3.6–7.0 μm, lateral, intercalary or terminal, globose to subglobose, somewhat ellipsoid, hyaline to dark brown, thick and smooth-walled, in short or singly chains.

**Figure 5.**
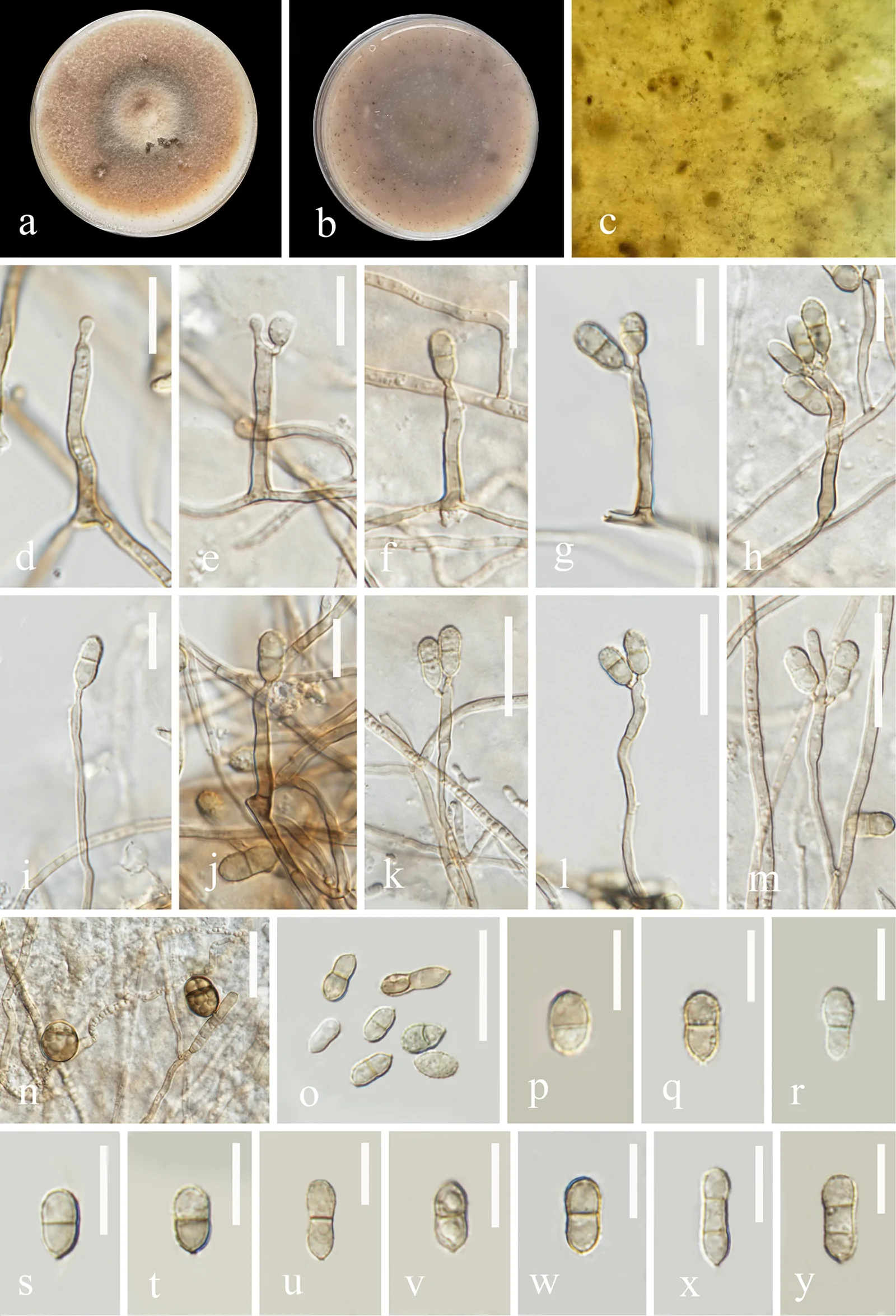
*Melnikomyces linderae* (HKAS 133201, holotype). **a, b**. Cultures on OA from above and below; **c**. Colonies on OA; **d–m**. Conidiogenous cells and conidia; **n**. Chlamydospores; **o–y**. Conidia. Scale bars: 10 μm (**d–j, p–y**); 20 μm (**k–o**).

##### Culture characteristics.

Colonies on PDA reaching 2.1 cm diam. after 14 d at 28 °C, flat, cottony to floccose, smooth margins, cream-colored to brown; reverse dark brown, margin cream-colored. On OA, cottony to floccose, dark brown to medium brown, with sparse to moderate aerial hypha; reverse brown.

##### Material examined.

China • Anhui Province, Huangshan City, Huangshan District, 30°1.74'N, 117°32.99'E, elevation 273 m, root of *L. aggregata*, 13 August 2023, Meng-Lan Chen, SRPZ15-7-1 (Holotype: HKAS 133201, ex-type culture: KUNCC24-17617, ex-isotype culture: KUNCC25-20169).

##### Notes.

*Melnikomyces linderae* features pale brown to brown, narrow conidiophores (8.2–46.0 × 0.9–1.7 μm) together with polyblastic conidiogenous cells that bear cylindrical denticles. The verrucose, 1(–2)-septate conidia are 3.0–6.4 × 1.8–2.7 μm and are released by rhexolytic secession; globose to subglobose chlamydospores are present. Phylogenetically, *M. linderae* formed a sister clade to *M. vietnamensis* (Fig. 2). A comparison of nucleotide base pairs between them revealed differences of 24/527 bp (4.55%, including 3 gaps) and 8/748 bp (1.06%, no gaps) in the ITS and LSU sequences, respectively. Morphologically, *M. linderae* (KUNCC24-17617) is distinguished from *M. thailandicus* (CBS 145767) and *M. vietnamensis* (CBS 136209) (Crous et al. 2014; Hernández-Restrepo et al. 2020). *Melnikomyces thailandicus* and *M. vietnamensis* have larger conidia than *M. linderae*. Those of *M. thailandicus* are smooth, fusoid, subhyaline, (8–)9.5–12(–13) × (2–)2.5(–3) μm, while those of *M. vietnamensis* are fusoid-ellipsoidal, (7–)9–10(–11) × (2.5–)3(–3.5) μm. Furthermore, *M. thailandicus* and *M. vietnamensis* have distinctly wider conidiophores (2.0–3.0 μm and 1.5–2.0 μm, respectively) than *M. linderae* (0.9–1.7 μm). Therefore, strains KUNCC24-17617 and KUNCC25-20169 are introduced herein as *M. linderae* sp. nov.

## Discussion

This study represents one of the first taxonomic investigations of root-associated fungi of *L. aggregata*. The isolation of two new species (*H. rhizosphaerae* from the rhizosphere soil and *M. linderae* from the root) and a new host record for *H. taylorii* reveals previously undocumented fungal diversity. These findings establish a taxonomic framework for future studies on the functional roles of root-associated fungi in *L. aggregata*.

The genera *Hawksworthiomyces* and *Melnikomyces* are relatively small, comprising only eight and three formally described species, respectively, and have been documented from a limited range of substrates and geographic regions. *Hawksworthiomyces* species are typically associated with decaying plant organic substrates and humus-rich habitats (De Beer et al. 2016; Tan et al. 2022; Crous et al. 2023; Liu et al. 2025; Tan et al. 2025), while *Melnikomyces* species have been reported predominantly from fallen branches, leaf litter, or forest debris (Crous et al. 2014; Hernández-Restrepo et al. 2020; Wei et al. 2020). The discovery of *H. taylorii* and *M. linderae* from living root tissues expands the known ecological ranges of both genera, yet the ecological significance of these associations remains unclear. Nonetheless, the results underscore the critical importance of systematically sampling understudied host plants and suggest that additional undescribed lineages likely exist within these genera.

The phylogenetic analysis reveals that the lineage previously designated as OTU2029 groups together with *H. rhizosphaerae* but is clearly distinct from the ex-type strain of *H. crousii* (CMW37531) (Voyron et al. 2022). This difference indicates that OTU2029 was likely misidentified in the earlier environmental DNA study. Such misidentifications are not uncommon when identifications rely solely on sequence similarity without corroborating evidence from living cultures or morphological examination. This study clarifies that this lineage represents a novel species (*H. rhizosphaerae*) by providing the first living cultures and detailed phylogenetic and morphological evidence.

Future studies should include systematic sampling across a wider range of host plants, substrates, and geographic regions to better delimit species boundaries within *Hawksworthiomyces* and *Melnikomyces*. The use of genome-scale data will also help clarify their evolutionary relationships and potential functional traits. In addition, the ecological roles of these fungi in root-associated ecosystems, as well as their potential interactions with the medicinal plant *L. aggregata*, deserve further investigation. Expanding sampling efforts and applying integrative taxonomic approaches may reveal additional hidden diversity within these genera.

## Supplementary Material

XML Treatment for Hawksworthiomyces rhizosphaerae

XML Treatment for Hawksworthiomyces taylorii

XML Treatment for Melnikomyces linderae

## Additional information

### Artificial Intelligence (AI) use

The authors accept full responsibility for the content of the manuscript, including the disclosure of any use of AI.

No AI tools were used in the preparation of this manuscript.

### Author ORCIDs

M.-L. Chen https://orcid.org/0000-0002-0095-2998

N. Mukjang https://orcid.org/0000-0002-7397-3845

X. Luo https://orcid.org/0000-0001-6206-8711

### Data availability

All sequences generated in this study are available in the GenBank repository. Specimens are deposited in the Herbarium of Cryptogams, Kunming Institute of Botany, Academia Sinica (HKAS), Kunming, Yunnan Province, China.
